# The Role of Indocyanine Near-Infrared Fluorescence in Colorectal Surgery

**DOI:** 10.3389/fsurg.2022.886478

**Published:** 2022-05-20

**Authors:** Francesco Maione, Michele Manigrasso, Alessia Chini, Sara Vertaldi, Pietro Anoldo, Anna D’Amore, Alessandra Marello, Carmen Sorrentino, Grazia Cantore, Rosa Maione, Nicola Gennarelli, Salvatore D’Angelo, Nicola D’Alesio, Giuseppe De Simone, Giuseppe Servillo, Marco Milone, Giovanni Domenico De Palma

**Affiliations:** ^1^ Department of Clinical Medicine and Surgery, Federico II University of Naples, Naples, Italy; ^2^ Department of Advanced Biomedical Sciences, Federico II University of Naples, Naples, Italy

**Keywords:** indocyanine green, colorectal cancer, perfusion, minimally invasive surgery, nearinfrared fluorescence

## Abstract

**Purposes:**

The aim of this study was to evaluate the importance of Indocyanine Green in control of anastomosis perfusion and on anastomotic leakage rates during laparoscopic and robotic colorectal procedures.

**Methods:**

A retrospective review of patients who underwent elective minimally invasive surgery for colorectal cancer from 1 January 2018 to 31 December 2020 was performed. All patients underwent Near-Infrared Fluorescence-Indocyanine Green system in two moments: before performing the anastomosis and after completing the anastomotic procedure. Primary outcomes were the rate of intraoperative change in the surgical resection due to an inadequate vascularization and the rate of postoperative anastomotic leakage. Secondary outcomes were the postoperative complications, both medical and surgical (intra-abdominal bleeding, anastomotic leakage).

**Results:**

Our analysis included 93 patients. Visible fluorescence was detected in 100% of the cases. In 7 patients (7.5%), the planned site of resection was changed due to inadequate perfusion. The mean extension of the surgical resection in these 7 patients was 2.2 ± 0.62. Anastomotic leakage occurred in 2 patients (2.1%). Other complications included 8 postoperative bleedings (8.6%) and 1 pulmonary thromboembolism.

**Conclusions:**

The intraoperative use of Near-Infrared Fluorescence-Indocyanine Green in colorectal surgery is safe, feasible, and associated with a substantial reduction in postoperative anastomotic leakage rate.

## Introduction

Anastomotic leakage (AL) is defined as a dehiscence of the intestinal wall at the anastomotic site, that could require a surgical revision, and it represents one of the most common complications in colorectal surgery. The incidence of AL in ileocolic, colo-colic, and colorectal or coloanal anastomoses is 1–4%, 2–3%, and 5–19%, respectively (1, 2). In most cases, the development of AL depends on the state of perfusion, the surgical technique, and the anastomotic procedure. Data reported from the literature showed that there is no difference in the AL rate between open surgery and minimally invasive techniques; regarding the anastomotic technique, anastomosis with stapling devices is associated with a higher incidence of AL with respect to non-stapled anastomosis, as confirmed by a recent study conducted by Wurtz et al (3). Complications following surgery can be due to technical errors such as insufficient blood supply and increased tension to the anastomosis, technical failure of the stapler, and inadequate suturing. Advances in technology have introduced near-infrared (NIR) fluorescence imaging with indocyanine green (ICG) to evaluate the perfusion of colorectal anastomosis.

The aim of this study was to evaluate the role of ICG in control of perfusion to the anastomosis and on AL rates during minimally invasive colorectal surgery.

## Material and Methods

After the approval of the Institutional Review Board of the “Federico II” University of Naples, a retrospective chart review of the minimally invasive colorectal resection for cancer from 1 January 2018 to 31 December 2020 was performed.

All patients received an elective laparoscopic or robotic operation and they underwent previously a complete history and physical examination with blood tests, cross-sectional imaging (4), and colonoscopy. After the admission, the patients underwent bowel preparation with a combination of osmotic laxative, potassium and sodium salts if possible, preoperative antibiotics, and heparin prophylaxis according to the current literature (5–8).

### Surgical Technique

All operations were performed by expert surgeons. In order to reduce the bias related to the different surgical techniques, only procedures performed according to the standardized criteria were included in the study.

All the patients were operated on under general anesthesia (9). In right colectomy, once identified the ileocolic pedicle, the peritoneum of the mesentery just inferior to the vessel should be opened with the creation of a mesenteric window. Thus, Toldt’s fascia was separated from Gerota’s plane, with identification and preservation of the right ureter, duodenum, and pancreatic head. After ligation of the ileocolic pedicles at their origin, the right colon was completely mobilized laterally from the right parietocolic gutter. The mesentery was dissected medially, with consequent ligation of the right colic vessels and the right branch of the middle colic vessels. After performing the right hemicolectomy with a linear stapler, the ileo-colic anastomosis was performed intracorporeally in a side-to-side isoperistaltic fashion. In the left colectomy, after the colo-epiploic detachment and the complete mobilization of the splenic flexure, the Inferior Mesenteric Vein (IMV) and the Inferior Mesenteric Artery (IMA) were isolated, clipped, and divided at their roots. After the detachment of the Toldt’s fascia from the Gerota’s plane, with the preservation of the retroperitoneal elements, left hemicolectomy was performed with a linear stapler and a colorectal end-to-end anastomosis was performed according to Knight–Griffen technique. In the case of anterior rectal resection, after the complete mobilization of the left colon as described, the intervention proceeded with a Partial or Total Mesorectal Excision (PME or TME). In segmental splenic flexure resection, after the mobilization of the descending and transverse colon, the left branches of the middle colic vessels and the left colic artery were isolated, clipped, and ligated at their origin. Finally, for transverse colon resection, both the colic flexures were completely mobilized and a wedge resection of the mesentery, including the branches of the middle colic artery, was performed. In the case of segmental resections, the colo-colic anastomosis was performed intracorporeally in a side-to-side isoperistaltic way.

**Figure 1:**
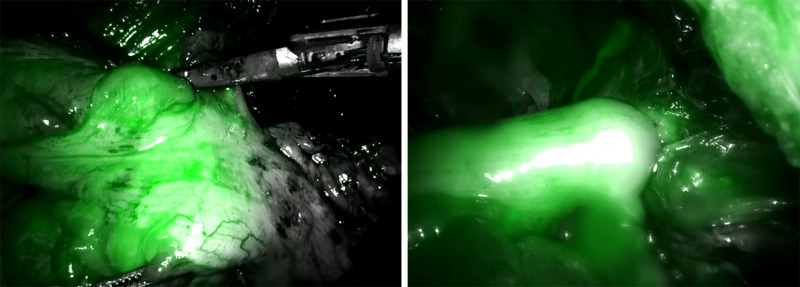
Pre- (**A**) and post-anastomotic (**B**) application of the Indocyanine Green technology.

All patients underwent NIR/ICG system according to a standardized technique at two different moments: before performing the anastomosis to control the adequate vascularization of the stumps and after completing the anastomosis to control its perfusion (Figure 1). In detail, before the colonic or rectal resection, the anesthesiologist administered a bolus of 0.2 mg/kg of ICG, and after a median time of 25 seconds, an adequate vascularization was visible (if present). The same procedure was repeated after performing the anastomosis.

### Data Collection and Outcomes Assessment

Data were prospectively collected and included gender, age, Body Mass Index (BMI), American Society of Anesthesiologists (ASA) Score, conversion rate, and intraoperative complications.

Primary outcomes included the rate of intraoperative change in the surgical resection due to an inadequate vascularization at the NIR/ICG system and the rate of postoperative anastomotic leakage. In case of intraoperative changes due to an inadequate vascularization, the extension of the surgical resection was measured in centimeters and registered.

Secondary outcomes were the postoperative complications according to the Clavien–Dindo classification.

Anastomotic leakage was suspected based on fever, abdominal pain, fecal matter in abdominal drainage, abscess and gas around the anastomotic site at the computed tomography, and the presence of a communication between inside and outside the intestinal tract at the contrast enema. Anastomotic leakage was considered as a complication when a surgical re-intervention was necessary.

### Statistical Analysis

Statistical analysis was performed using the SPSS 26 system (SPSS Inc., Chicago, IL, USA). Continuous data were expressed as mean ± SD; categorical variables were expressed as %. Furthermore, a multivariate analysis was performed to assess if any patients’ or surgical characteristics (age, gender, BMI, ASA Score, the presence of diabetes or hypertension, the adoption of robotic or laparoscopic approach) could significantly impact on anastomotic leakage or bleeding rate. Results of the multivariate analysis were expressed by Odds Ratio (OR) and 95% Confidence interval (95% CI).

## Results

Our analysis included 93 patients; all patients underwent elective minimally invasive surgery (laparoscopic or robotic surgery) for malignant colorectal cancer. Of these, 40 were female and 53 were male (43% and 57%, respectively).

Demographic data are reported in Table 1. Mean age was 69.81 ± 12.06, mean BMI was 25.81 ± 4.29, and mean ASA Score was 2.54 ± 0.52. Of the included patients, 16 (17.2%) were smokers, 12 (12.9%) were obese, 20 (21.5%) were affected by diabetes, and 56 (60.2%) by hypertension. 24 patients (25.8%) underwent previous abdominal surgery.

**Table 1:** Demographic data of the included patients.

Patients (*N*)	93
Gender	
M	53 (57)
F	40 (43)
Age (years)	69.81 ± 12.06
BMI	25.81 ± 4.29
ASA score	2.54 ± 0.52
I	1 (1)
II	41 (44.1)
III	51 (54.9)
Smokers	16 (17.2)
Comorbidity	
Hypertension	56 (60.2)
Diabetes	20 (21.5)
Obesity	12 (12.9)
Previous abdominal intervention	24 (25.8)

*Categorical variables are expressed as number and (percentage); continuous variables are expressed as mean ± standard deviation (SD). BMI, Body Mass Index.*

Intraoperative data are reported in Table 2. Of the included procedures, 62 (66.6%) were laparoscopic and 31 (33.4%) robotic. 41 were right hemicolectomy (44.1%), 24 (25.8%) left hemicolectomy, 23 (24.7%) were rectal anterior resection (with or without protective loop ileostomy), 4 (4.3%) splenic flexure resection, and 1 (1.1%) was a segmental resection of the transverse colon. Intraoperative complications included 3 intraoperative bleeding, with no conversion needed. After the injection of the ICG, no adverse events were registered.

**Table 2:** Intraoperative data.

**Intraoperative data**	*N*
Surgical technique	
Laparoscopic	62 (66.6)
Robotic	31 (33.4)
Type of resection	
Right hemicolectomy	41 (44.1)
Left hemicolectomy	25 (25.8)
Rectal anterior resection	23 (24.7)
Splenic flexure resection	4 (4.3)
Transverse colon resection	1 (1.1)
Intraoperative complications	3
Intraoperative bleeding	3 (3.2)
Conversion	0
ICG characteristics	
Detection	93 (100)
Change in planned resection	7 (7.5)
Extension of the modified surgical resection	2.2 ± 0.62

*Categorical variables are expressed as number and (percentage). Continuous variables are expressed as means ± standard variation (SD). ICG, Indocyanine Green.*

Visible fluorescence was detected in 100% of the cases and the meantime from ICG injection to visible fluorescence was 25 seconds. In 7 patients (7.5%), the planned site of resection was changed due to inadequate perfusion at NIR/ICG system. The mean extension of the surgical resection in these 7 patients was 2.2 ± 0.62.

After performing the anastomosis, the NIR/ICG system detected no cases of inadequate perfusion in the performed anastomoses.

Postoperative complications are summarized in Table 3. Anastomotic leakage occurred in 2 patients (2.1%), in which a protective loop ileostomy was performed. Other complications included 8 postoperative bleedings (8.6%), which required blood transfusion, and 1 pulmonary thromboembolism, which required implementation of anticoagulant therapy. Postoperative complications are defined according to the Clavien–Dindo classification, which consists of five severity grades. Grade 1 includes minor postoperative complications, not requiring therapy and none of the patients enrolled in the study is included in this group. Grade 2 complications require pharmacological treatment and eight patients with intra-abdominal bleeding are included in this group. Grade 3 complications, requiring surgical or radiological intervention, are recorded in 2 patients with anastomotic leakage, while Grade 4 complications, requiring an intensive care management for single or multiorgan disfunction, are recorded in 1 patient with pulmonary thromboembolism. Grade 5 indicates the death of the patient and it did not occur in any patients enrolled in the study (10).

**Table 3:** Postoperative complications.

Postoperative complications	*N*
Clavien–Dindo Classification	
I	0
II	8 (8.6)
Intra-abdominal bleeding	8 (8.6)
III	
Anastomotic leakage	2 (2.1)
IV	1 (1.1)
Pulmonary thromboembolism	1 (1.1)
V	0

*Categorical variables are expressed as number and (percentage).*

Table 4 reported results classifying patients according to precise categories: sex, age, obesity, defined as BMI > 30, presence of diabetes, hypertension, and previous abdominal intervention. For each category, the surgical technique, intraoperative complications, postoperative complications, and ICG detection were reported.

**Table 4:** Surgical technique, complications, and ICG detection according to patient categories.

	*N*	Laparoscopic surgery	Robotic surgery	Intraoperative complications	Postoperative complications	ICG detection
Male	53	37 (70%)	16 (30%)	3 (5.7%)	9 (17%)	53 (100%)
Female	40	25 (62.5%)	15 (37.5%)	0 (0%)	2 (5%)	40 (100%)
Age >60 y.o.	73	49 (67.1%)	24 (32.9%)	3 (4.1%)	8 (11%)	73 (100%)
Age >60 y.o.	20	13 (65%)	7 (35%)	0 (0%)	3 (15%)	20 (100%)
Obesity	12	8 (66.7%)	4 (33.3%)	0 (0%)	1 (8.3%)	12 (100%)
No obesity	81	54 (66.7%)	27 (33.3%)	3 (3.7%)	10 (12.3%)	81 (100%)
Diabetes	20	17 (85%)	3 (15%)	0 (0%)	3 (15%)	20 (100%)
No Diabetes	73	45 (61.6%)	28 (38.4%)	3 (4.1%)	8 (11%)	73 (100%)
Hypertension	56	33 (58.9%)	23 (41.1%)	2 (3.6%)	9 (16.1%)	56 (100%)
No hypertension	37	29 (78.4%)	8 (21.6%)	1 (2.7%)	2 (5.4%)	37 (100%)
Previous abdominal intervention	24	15 (62.5%)	9 (37.5%)	1 (4.2%)	3 (12.5%)	24 (100%)
No previous abdominal intervention	69	47 (68.1%)	22 (31.9%)	2 (2.9%)	8 (11.6%)	69 (100%)

*Categorical variables are expressed as number and (percentage). ICG: Indocyanine Green; y.o: years old.*

Table 5 showed the results of the multivariate analysis. Specifically, none of the patients’ characteristics or the adoption of robotic or laparoscopic approaches significantly impacted on the rate of anastomotic leakage and bleeding.

**Table 5:** Results of the multivariate analysis.

Characteristics	Leakage OR and (95% CI)	Leakage *p*-value	Bleeding OR and (95% CI)	Bleeding *p*-value
Gender	0.825 (0.037–18.416)	0.903	1.467 (0.312–6.903)	0.628
Age	0.942 (0.820–1.081)	0.396	1.002 (0.929–1.080)	0.963
BMI	1.214 (0.713–2.066)	0.475	0.966 (0.821–1.136)	0.675
Diabetes	4.889 (0.11–216.793)	0.412	0.983 (0.154–6.271)	0.986
Hypertension	0.802 (0.031–20.704)	0.894	0.843 (0.167–4.252)	0.836
ASA Score	2.610 (0.101–67.395)	0.563	0.535 (0.081–3.532)	0.516
Robotic/laparoscopic intervention	0.842 (0.070–10.186)	0.892	0.814 (0.237–2.801)	0.744

## Discussion

Although the benefits of the minimally invasive surgery in the treatment of the colorectal pathologies are well known (11–15), anastomotic leakage (AL) remains one of the most common complications in colorectal surgery. It increases morbidity and mortality, healthcare costs, and worsening long-term oncological outcomes. The risk factors for AL include: preoperative findings, such as tumor size and stage (16, 17), radiation, chemotherapy, male sex (18), nutrition (17), and comorbid condition such as obesity (19, 20), diabetes mellitus, cardiovascular disease; intraoperative factors, including the state of bowel perfusion, the level and the tension of anastomosis (21, 22), blood loss and operation time (16, 19, 23, 24); postoperative factors, such as the presence of diverting stoma (21), placement of abdominal drainage tube (16), and changes of intestinal microbes (25). Malignant involvement of local mesenteric lymph nodes could lead to mesenteric lymphadenopathy and increase the risk of complications, including AL, as reported also for several types of tumors (26).

The surgical technique and the state of perfusion are known to be important factors for the occurrence of AL. For years, anastomotic blood perfusion is assessed by the surgeons with visual evaluation of the resection margins, even if it has been reported to be a subjective analysis. In the last years, several studies have suggested that NIR has emerged as a promising method for a more accurate assessment of tissue perfusion during colorectal surgery, following intravenous infusion of indocyanine green (ICG).

ICG is a tricarbocyanine compound with a molecular mass of 776 Da, soluble in water. After its intravenous injection, ICG is quickly fixed to plasmatic proteins and, from the blood circulation, is carried to the liver, where ICG is extracted unchanged. In the case of extravenous injection, ICG is found in macrophages located in lymphatic vessels and lymph nodes.

ICG is captured by a system that activates its fluorescence with the light emitted by a led. Once excited, ICG sends fluorescent signals that have the ability to cross about 10 mm of the human soft tissue. From the intravenous injection, the spread of ICG to peripheral vessels is very rapid, in terms of few seconds. The reduction of the blood flow in a tissue leads to a decrease in ICG fluorescence emission. The evaluation of blood perfusion using ICG fluorescence imaging is applied not only to colorectal resection but also to breast reconstruction and coronary artery bypass grafting.

Several studies have reported the efficacy and the feasibility of the ICG injection in patients who underwent different surgical interventions under election for colorectal cancer (1, 27–33).

Impellizzeri et al. (1) showed that the intraoperative use of NIR/ICG for evaluation of anastomosis perfusion was safe for colorectal surgery and it significantly reduces the AL incidence. They conducted a retrospective study including 196 procedures of which 98 were without the use of ICG imaging and 98 were with the use of ICG imaging. In the first group, six patients developed AL, in the second no one. Similar encouraging results have been shown by other studies. Boni et al. (27) and Jafari et al. (28) conducted two case-control analyses, showing that the use of NIR/ICG for low anterior rectal resections, where the risk of AL is higher than in other large intestinal resection, demonstrated inadequate blood perfusion on the anastomosis site in 5%–19% of cases, thus the colonic transection point was changed, and it was associated with a reduction in AL rate (5%–12%), in comparison to the control group. The use of NIR/ICG in right and left hemicolectomy, segmental resection and anterior rectal resection reported a change of section line in 3.7%–7.9% of cases following NIR/ICG, with an AL incidence of 0.9%–1.4% (34, 35). Morales-Conde et al. (29), in their study, enrolled 192 patients who underwent different colorectal surgical procedures to evaluate in which one fluorescence angiography with indocyanine green (ICG-FA) was more effective in the anastomosis assessment, changing the section line level. The most significant value was observed in left hemicolectomy (25.9%), followed by anterior rectal resection (25.7%), segmental resection of the splenic flexure (11.1%), and right hemicolectomy (6%). Hasegawa et al. (2) conducted a retrospective study on 844 patients who underwent laparoscopic sphincter-sparing surgery: among them, 141 patients underwent ICG-FA to identify AL, and they were compared to 703 patients in whom ICG-FA was not performed. The incidence of AL was 2.8% in the first group and 12.4% in the second one. Also, Ishii et al. (31) evaluated the role of ICG-FA in their retrospective analysis, including 488 patients with colorectal cancer who underwent surgical intervention. ICG-FA was performed in 233 patients and they showed that the incidence of AL was no significantly different between the two groups in patients with colon cancer, while, in patients with rectal tumor, the incidence of AL was lower in the ICG group than in the no-ICG group (3.5% vs 10.5%). The retrospective case–control study by Brescia et al. (32) confirmed that the use of ICG-FA in patients managed with ERAS perioperative protocol was feasible, safe, and reduced the anastomotic leakage. They enrolled 182 patients who underwent laparoscopic colorectal surgery and divided them into two groups: a first group (A) including 107 patients managed with ERAS perioperative protocol and a second group (B) including 75 patients managed not only with ERAS pathway but also with the use of ICG-FA. 6 (5.6%) clinically relevant AL occurred in group A while there was none in group B. In a retrospective study, Kin et al. (33) evaluated the use of ICG-FA for the assessment of anastomosis perfusion in patients underwent colorectal surgery, but they did not find any advantage from the use of NIR/ICG, showing that the pelvic radiation therapy and the anastomosis proximity from anal verge were independent predictors of AL. However, they evaluated with NIR/ICG only the proximal point of transection. Thus, we believe that to reduce the AL rate, it is important to evaluate with NIR/ICG the perfusion of both the transection point and of the anastomosis once completed.

However, there is no unified system for the quantitative analysis of the fluorescence signal, thus it is not possible to reproduce and compare results from various studies. Moreover, there are some technical aspects of fluorescence imaging that we have to consider. Firstly, fluorescence intensity depends on the distance between the emission source and the target tissue. Moreover, ICG circulates dynamically in the tissues according to perfusion and this often leads to an underestimation of ischemic zones. Actually, few studies regarding the quantitative evaluation of perfusion in the colorectal anastomotic site are reported in literature. One of them is the study by Amagai et al. (36), where authors, in the evaluation of intestinal perfusion during colorectal surgery, considered four areas of interest: two proximal intestinal areas, one where the fluorescence was higher (proximal-high) and the other where it was lower (proximal-low), and two distal intestinal areas, one where the fluorescence was higher (distal-high) and the other where it was lower (distal-low). In each area, they considered the time from the intravenous injection of ICG to the maximum fluorescence (*T*_max_) and the time from the start of dyeing to the *T*_max_, which is defined as Δ*T*, and they found a correlation between *T*_max_ e Δ*T*. Wada et al. (37) showed a correlation between the maximum fluorescence value (Fmax) and AL. On the contrary, Hayami et al. (38), analyzing Fmax data in their results, hypothesized a correlation with breath excursions, especially in minimally invasive surgery. For this reason, they considered Fmax as an unstable factor and it cannot be considered a feasible indicator of AL. Thus authors focused on the relationship between the period from the intravenous infusion of ICG to the beginning of fluorescent emission (T0) and AL and showed that patients with AL had longer T0 than those without AL. In another report, Son et al. (39) showed a correlation between time from first fluorescence increase to half of the maximum and AL and a correlation between the time ratio (time from first fluorescence increase to half of the maximum/ the time from the start of dyeing to the maximum fluorescence) and AL. D’Urso et al. (40) demonstrated that fluorescence-based enhanced reality (FLER) can be an accurate method to quantify fluorescence signal in augmented reality and to provide a feasible evaluation of intestinal perfusion.

During colorectal resections, ICG imaging also provides to facilitate vascular dissection when the vascular anatomy of tumor site is unclear and identifies the ureter to prevent iatrogenic injury. Santi et al. (41) prospectively enrolled 38 patients for a standard surgical treatment of laparoscopic colorectal resection, in six cases they used ICG imaging to identify vascular anatomy and to perform vascular dissection, in one case they used ICG imaging to identify the ureter which was tightly attached to the tumor.

In our study, we use indocyanine near-infrared fluorescence in two moments: before performing the anastomosis to control transection points and after completing the anastomotic procedure to control its perfusion. In 7.5% of the cases, the planned site of transection was changed because the demarcation line defined by NIR/ICG system was different from the point established by the surgeon’s visual inspection. AL developed only in 2.1% of the cases. In conclusion, the results of this retrospective analysis suggest that the intraoperative use of NIR/ICG in colorectal surgery is safe and feasible, both before performing the anastomosis to control the site of resection than after performing the anastomosis to evaluate the perfusion of the anastomotic site, regardless of patients’ characteristics and the surgical approach. Furthermore, the use of ICG did not result in allergic reactions and prolonged surgery time, and postoperative complications were not consequential to the additional technique. However, major limitations of this study have to be addressed. Being a retrospective cohort analysis, the bias related to the absence of randomization and of a control group clearly constituted a concern. For this reason, randomized prospective trials on intraoperative NIR/ICG use are necessary to confirm these data.

Therefore, despite there are several questions to be discussed and more high-quality large sample size randomized prospective trials are necessary to confirm the benefits of NIR/ICG in colorectal surgery, we believe that the assessment of an adequate vascularization by the use of NIR/ICG should be considered a key point to reduce the incidence of AL.

## Data Availability

The raw data supporting the conclusions of this article will be made available by the authors, without undue reservation.
